# Stochastic variational inference improves quantification of multiple timepoint arterial spin labelling perfusion MRI

**DOI:** 10.3389/fnins.2025.1536752

**Published:** 2025-02-04

**Authors:** Thomas F. Kirk, Georgia G. Kenyon, Martin S. Craig, Michael A. Chappell

**Affiliations:** ^1^Quantified Imaging Limited, London, United Kingdom; ^2^Sir Peter Mansfield Imaging Centre, School of Medicine, University of Nottingham, Nottingham, United Kingdom; ^3^School of Computer and Mathematical Sciences, University of Adelaide, Adelaide, SA, Australia

**Keywords:** perfusion, arterial transit time (ATT), arterial spin label (ASL) MRI, cerebral blood flow (CBF), quantification

## Abstract

Multiple-timepoint arterial spin labelling MRI is a non-invasive imaging technique that permits measurement of both cerebral blood flow and arterial transit time, the latter of which is an emerging biomarker of interest for cerebrovascular health. Quantification of arterial spin labelling data is challenging due to the low signal to noise ratio and non-linear tracer kinetics of this technique. In this work, we introduce a new quantification method called SSVB that addresses limitations in existing methods and demonstrate its performance using simulations and acquisition data. Simulations showed that the method is more accurate, particularly for estimating arterial transit time, and more robust to noise than existing techniques. On high spatial resolution data acquired at 3 T, the method produced less noisy parameter maps than the comparator method and captured greater variation in arterial transit time on a cross-sectional cohort.

## Introduction

Arterial spin labelling (ASL) is a non-invasive magnetic resonance imaging (MRI) technique that permits the measurement of perfusion in the brain. Using strong magnetic gradient fields applied for a short time across the neck, inflowing blood-water is magnetically inverted shortly before an image of the brain is acquired, after which tracer kinetic modelling can be used to quantify the rate and speed of blood flow (Chappell et al., 2017; Buxton et al., 1998). By capturing the dynamics of inflowing label, multiple-timepoint ASL permits the measurement of both cerebral blood flow (CBF) and arterial transit time (ATT), the latter of which is an emerging marker of cerebrovascular health, for example associated with Alzheimer’s disease and coronary artery disease (Nielsen et al., 2017; MacIntosh et al., 2015). Estimating ATT from multiple-timepoint arterial spin labelling (ASL) data is however challenging due to the non-linear signal model based on tracer kinetics and the inherently low signal-to-noise ratio (SNR) of this imaging technique (Chappell et al., 2017). This is particularly true in white matter, which has lower CBF and longer ATT than cerebral grey matter (thus reducing SNR), but is implicated in a range of neurological disorders such as schizophrenia and vascular dementia (Wright et al., 2014; Barker et al., 2014).

Existing methods for ATT estimation adopt a number of strategies to deal with this challenge. Signal-weighted delay (WD) approximates ATT using a first-order moment calculation that is simple to implement and robust to noise, though the accuracy of the approximation is bounded by the choice of post label delays (PLD) used and it is unclear how to adapt the method to handle variable label durations, as used for example in Hadamard-encoded schemes in which multiple boluses are delivered (Dai et al., 2012; von Samson-Himmelstjerna et al., 2016). Non-linear least squares (NLLS) is widely available in software libraries but is vulnerable to noise, for which reason the data is commonly smoothed in pre-processing to increase SNR, or hard constraints on ATT may be implemented. FSL BASIL adopts a variational Bayesian (VB) approach that offers a more principled treatment of low SNR data via the use of priors, but this requires advance knowledge of the normative range of ATT which may not be available in certain populations (Chappell et al., 2023).

In this work we introduce a new method for perfusion estimation on multiple-timepoint ASL data and demonstrate its performance on both simulation and acquisition data. Compared to existing methods, Structured Stochastic Variational Bayes (SSVB) is a VB method that produces more accurate estimates, is more robust on low SNR data, and produces less noisy parameter maps.

## Theory

Bayesian inference is an attractive strategy for model-fitting ASL data because it allows parameter constraints to be implemented in a principled manner (mitigating against low SNR) and quantifies the uncertainty of the resulting estimates (Chappell et al., 2009; Woolrich et al., 2009). Bayesian inference seeks to obtain the posterior distribution on model parameters conditioned on the modality specific signal model and the data that has been acquired. For all but the simplest problems, derivation of the true posterior is generally intractable due to the integrals that must be performed. Variational Bayesian (VB) techniques circumvent this problem by approximating the posterior using a simpler distribution with well-known properties, which is optimised to make the approximation as accurate as possible (Penny et al., 2003). Specifically, VB seeks to minimise the Kullback–Leibler divergence between the approximate and true posterior, which is equivalent to maximising the free energy


$$F\left(\theta \right)=\int q\left(\theta \right)\log\left(p\left(y|\theta \right)\frac{p\left(\theta \right)}{q\left(\theta \right)}\right)d\theta$$

Where θ represents the physiological parameters of interest, y the samples of data, $q\left(\theta \right)$ the approximating posterior distribution, $p\left(y|\theta \right)$ the likelihood, and $p\left(\theta \right)$ prior distribution for the parameters. Noise is explicitly modelled in the likelihood, typically as a zero-mean Gaussian distribution. Qualitatively, this expression can be seen as maximising the log-likelihood (a best fit to the data) whilst minimising the divergence of the approximating posterior from the priors which enforces the constraints on parameter estimates. Priors can be either distributional, for example a Gaussian of specified mean and variance; or spatial, which in the context of imaging encodes the belief that neighbouring voxels should not differ substantially from each other. Spatial priors have a similar effect to spatial smoothing which is commonly used in neuroimaging but have the key advantage of being able to determine the optimal amount of smoothing from the data itself rather than requiring the user to specify a value (Chappell et al., 2011; Penny et al., 2005).

FSL BASIL is an existing implementation of VB for ASL that uses an analytic formulation in which the signal model is linearised via a Taylor expansion, after which iterative update equations are derived via the calculus of variations (Chappell et al., 2009; Chappell et al., 2023). Though computationally fast, this approach has a number of drawbacks: firstly, in order to keep the derivation of update equations tractable, the parametric distributions that are used to represent the prior and posterior are generally restricted to the exponential family (e.g., Gaussian, often referred to as the ‘conjugate-exponential’ restriction), which may not be appropriate in a particular application. Secondly, should the analytic form of priors or noise model change, it is necessary to re-derive the update equations from scratch.

In recent years, the increasing use of neural networks has been accompanied by improvements in optimisation techniques based on fast and efficient gradient descent which have been released to the public in libraries such as TensorFlow and PyTorch (Abadi et al., 2016; Paszke et al., 2019). The availability of these alternative optimisation techniques offers a new possibility for implementing VB, namely, direct optimisation of the expression for free energy. Specifically, using a Monte Carlo sampling technique, an approximation for $F\left(\theta \right)$ may be obtained by drawing L samples of θ , the physiological parameters of interest, from the posterior $q\left(\theta \right)$ , where * denotes a sample.


$$F\left(\theta \right)\approx \frac{1}{L}\overset{L}{\underset{l}{\sum }}\left[\log\left(p\left(y|\theta^{*l}\right)\right)-\log\left(\frac{q\left(\theta^{*l}\right)}{p\left(\theta^{*l}\right)}\right)\right]$$

This is the objective function for SSVB that is directly optimised via gradient descent [a more detailed derivation is given in (Chappell et al., 2020)]. The first term approximates the log-likelihood, encouraging a good model fit to the data, and the second term penalises divergence of the model parameters from the prior. In this work, a multi-variate normal distribution over CBF and ATT has been used without covariance, and zero-mean Gaussian noise is assumed (the noise variance is a parameter of the model). Under these assumptions, the log-likelihood may be expressed as


$$\log p\left(y|\theta \right)=\frac{N}{2}\log\phi -\frac{1}{2}\phi {\left(y-M\left(\theta \right)\right)}^{T}\left(y-M\left(\theta \right)\right)$$

where N is the number of observations (number of ASL images that have been acquired), ϕ is the noise precision (inverse variance) and the latter term represents the sum of squared differences between the model prediction for the current parameters θ and the acquired data y .

Within SSVB, a spatial prior is used to implement adaptive (data-driven) smoothing of the parameter maps, which is beneficial to mitigate against the low SNR of ASL. A first-order graph Laplacian operator is used, represented by the v x v matrix D where v is the number of voxels, off-diagonal elements are 1 if the corresponding row and column voxels are adjacent, and the diagonal elements are the negative sum of that voxel’s number of direct neighbours (−6 in the case of a fully-connected voxel). The prior for a single model parameter $\theta_{i}$ may then be expressed as


$$\log p\left(\theta_{i}\right)=\frac{1}{2}\log\alpha -\frac{\alpha }{2}\theta_{i}^{T}D\theta_{i}$$

Where α represents the spatial precision, the weight given to the spatial prior, and is a target of the optimisation itself. This means the amount of smoothing is automatically determined from the data and need not be specified by the user, which should better preserve spatial detail and generalise well across datasets without requiring hand-tuning, as has been observed in previous work (Zhao et al., 2017; Chappell et al., 2011).

The stochastic approach offers a number of advantages over analytic VB. For example, prior and posterior distributions no longer need to be drawn from the same family which removes the conjugate-exponential restriction. Secondly, because the free energy is optimised directly, it is no longer necessary to derive update equations which permits greater flexibility in modifying priors or noise models between different applications.

The ASL model used in this work is the Buxton single-compartment pseudo-continuous ASL (PCASL) model (Buxton et al., 1998), in which the magnetisation signal arriving in a voxel may be expressed as piecewise function


$$M\left(t\right)=\;\left\{\begin{array}{ll} 0 & for\;t<\Delta t\\ 2M_{0a}fT_{1\mathrm{app}}\left(1-e^{-\frac{t-\Delta t}{T_{1\mathrm{app}}}}\right) & for\;\Delta t\leq t<\Delta t+\tau \\ 2M_{0a}fT_{1\mathrm{app}}e^{-\frac{\Delta t}{T_{1b}}}e^{-\frac{t-\Delta t-\tau }{T_{1\mathrm{app}}}}\left(1-e^{-\frac{\tau }{T_{1\mathrm{app}}}}\right) & for\;\Delta t+\tau \leq t \end{array}\;\right.$$

Where 1T1app=1T1+fλ , t is the time since of the start of label creation (magnetic inversion of blood), f is perfusion, Δt is arterial transit time, $M_{0a}$ is the equilibrium magnetisation of arterial blood, $T_{1}$ and $T_{1b}$ are the longitudinal relaxation times for tissue and blood water respectively, and τ is the label duration.

## Methods and materials

### Datasets

#### Simulation data

**Figure 1:**
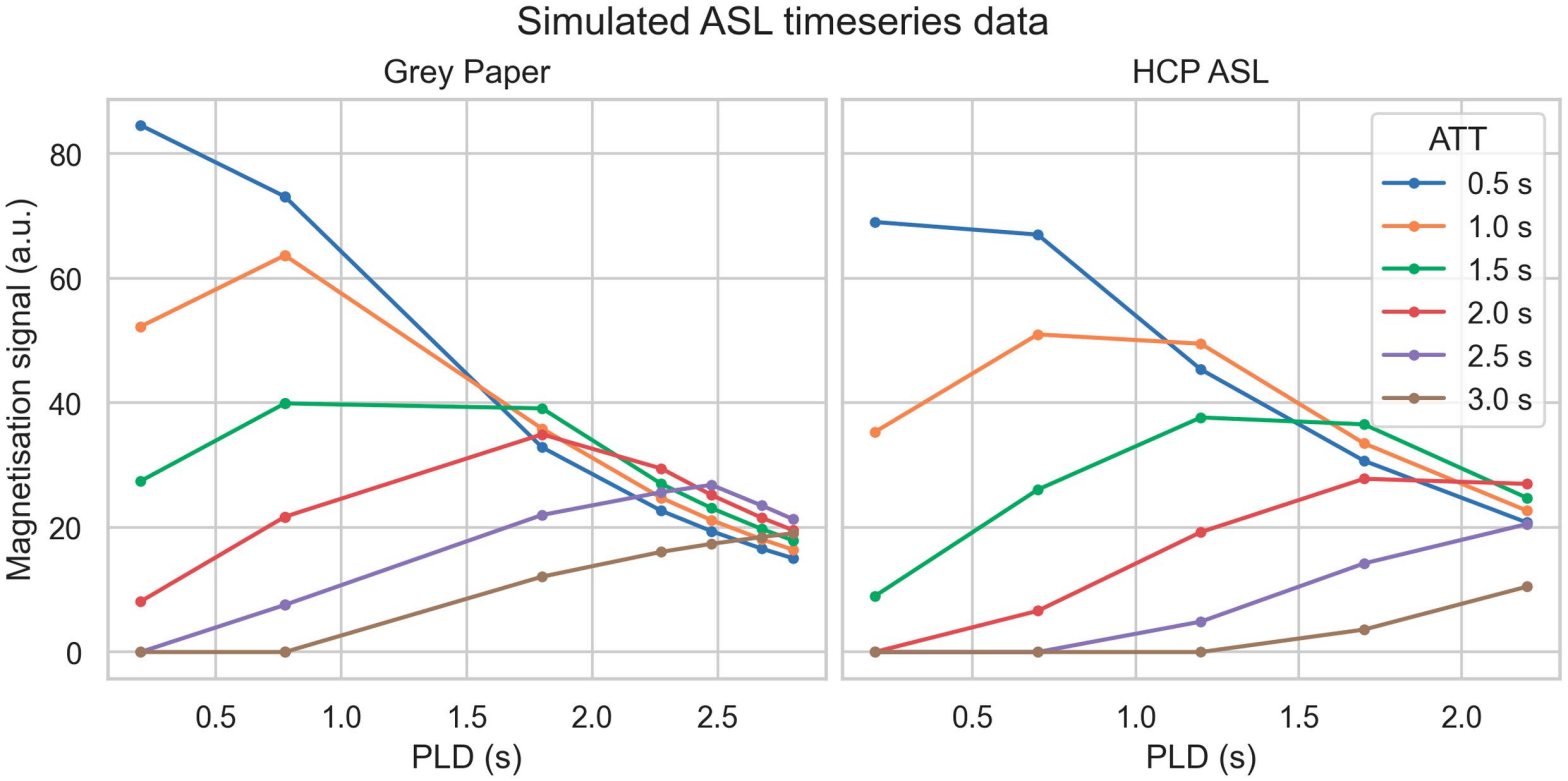
Simulated PCASL signal curves from each sequence (without noise) for a variety of ground truth ATT.

Two simulation datasets were used. The first of these (“grey paper”) used the optimal sampling scheme proposed by Woods et al. (2024) for perfusion measurement when ATT is expected to be in the range of 0.5 to 2.5 s specifically a 2.05 s label duration, 9 PLDs of 0.200, 0.775, 0.775, 0.775, 1.800, 2.275, 2.475, 2.675, 2.800 s with 4 repeats at each PLD. The second (“HCP ASL”) used the sampling scheme of the HCP Lifespan ASL dataset detailed in the following paragraph, specifically a 1.5 s label duration at 5 PLDs of 0.2, 0.7, 1.2, 1.7 and 2.2 s with 6, 6, 6, 10 and 15 repeats, respectively. For both datasets, data was simulated on an isotropic voxel grid of 5 voxels in each dimension. Ground truth CBF was held constant at 60 units whilst ATT was varied from 0.5 to 3.0 s inclusive in steps of 0.25 s. Zero-mean Gaussian noise with standard deviations (SD) of 10 to 40 inclusive in steps of 10 was added to each dataset to represent varying SNR. CBF was not varied because the PCASL signal scales linearly with CBF and would thus be equivalent to varying SNR. Noiseless signal curves for a range ground truth ATT values from each sequence are shown in Figure 1.

#### HCP ASL

The Human Connectome Project (HCP) Lifespan ASL dataset contains high spatial resolution multiple-timepoint ASL for over 3,000 subjects aged between 5 and 100+ years; a subset of 90 subjects (51 female, mean 31 years old, minimum 8 and maximum 88) was used in this work. This dataset was used because it used a sampling schedule specifically designed to capture ATT, should contain a wide range of ATT [a parameter that is known to increase with age (Feron et al., 2024)] and has a relatively challenging SNR (a consequence of the high spatial resolution), both of which make it difficult to accurately estimate perfusion. ASL data was acquired with the aforementioned sampling scheme at 2.5 mm isotropic resolution on a Siemens Prisma 3 T scanner. A calibration image was acquired at the same resolution with 8 s TR. T1 anatomical scans were also acquired at 0.8 mm isotropic. Further details of the acquisition are given in (Harms et al., 2018).

HCP ASL data was pre-processed using the HCP ASL minimal pre-processing pipeline version 0.1.5.post14 (stages 0 to 6 inclusive) to correct acquisition-related geometric and intensity artefacts and register the ASL/calibration images to the anatomical images (Kirk et al., 2024). Anatomical images were pre-processed using the HCP structural minimal processing pipelines, during which structural segmentation was performed using FreeSurfer (Glasser et al., 2013; Fischl, 2012). After perfusion estimation, reference region calibration was performed using the mean M0 value of ventricular CSF (Pinto et al., 2020).

### Inference methods

SSVB version 0.2.0 running on Python 3.11 using TensorFlow 2.16 was used for stochastic inference. A spatial prior was used on both CBF and ATT. RMSProp with a learning rate of 0.1 was used for optimisation (Ruder, 2017). After 50 successive steps without a decrease in cost, optimisation was reverted to the previous best state and the sample size increased by 1 from an initial starting size of 2; optimisation was run until 5 such reversions had taken place (typically around 1,000 epochs on a complete image, taking about 5 min with 8GB of RAM, or approximately twice as long as FSL BASIL).

Three comparator methods were used. FSL BASIL (“BASIL”) was used for conventional analytic VB inference, with a spatial prior for CBF whereas a normal distribution prior with mean 1.3 s and standard deviation 0.5 s was used on ATT (Chappell et al., 2009; Chappell et al., 2023). Signal weighted decay (“WD”) was implemented in two stages: first, using the first moment calculation to obtain ATT estimates and then providing these as fixed values to FSL BASIL to estimate CBF (Dai et al., 2012). Non-linear least squares (NLLS) was implemented using the FSL FABBER tool with –*method = nlls*. No constraints were implemented on either CBF or ATT, nor was any data pre-processing used (for example spatial smoothing). Like SSVB, BASIL explicitly models acquisition noise using a zero-mean Gaussian, whilst NLLS and WD make this assumption implicitly. On the basis of simulation results, only SSVB and BASIL were run on the HCP ASL acquisition data.

### Evaluation methods

For the simulation datasets, the mean across voxels of CBF and ATT estimates produced by each method was calculated, and the bias with respect to ground truth expressed as a percentage of ground truth.

For the HCP ASL dataset, the FreeSurfer DKT segmentation labels were grouped into five major cortical structures (frontal, temporal, parietal, occipital and cingulate) and the median CBF/ATT estimate reported within each by resampling the respective parameter maps onto the same voxel grid using trilinear interpolation. Methodological differences in parameter estimates were analysed using paired *t*-tests. Linear regression was used to investigate the relationship between age and CBF or ATT. Statistical significance for all tests was set at *p* = 0.05 and correcting for multiple comparisons between the five cortical structures reduced the threshold to *p* = 0.01.

## Results

### Simulation data

**Figure 2:**
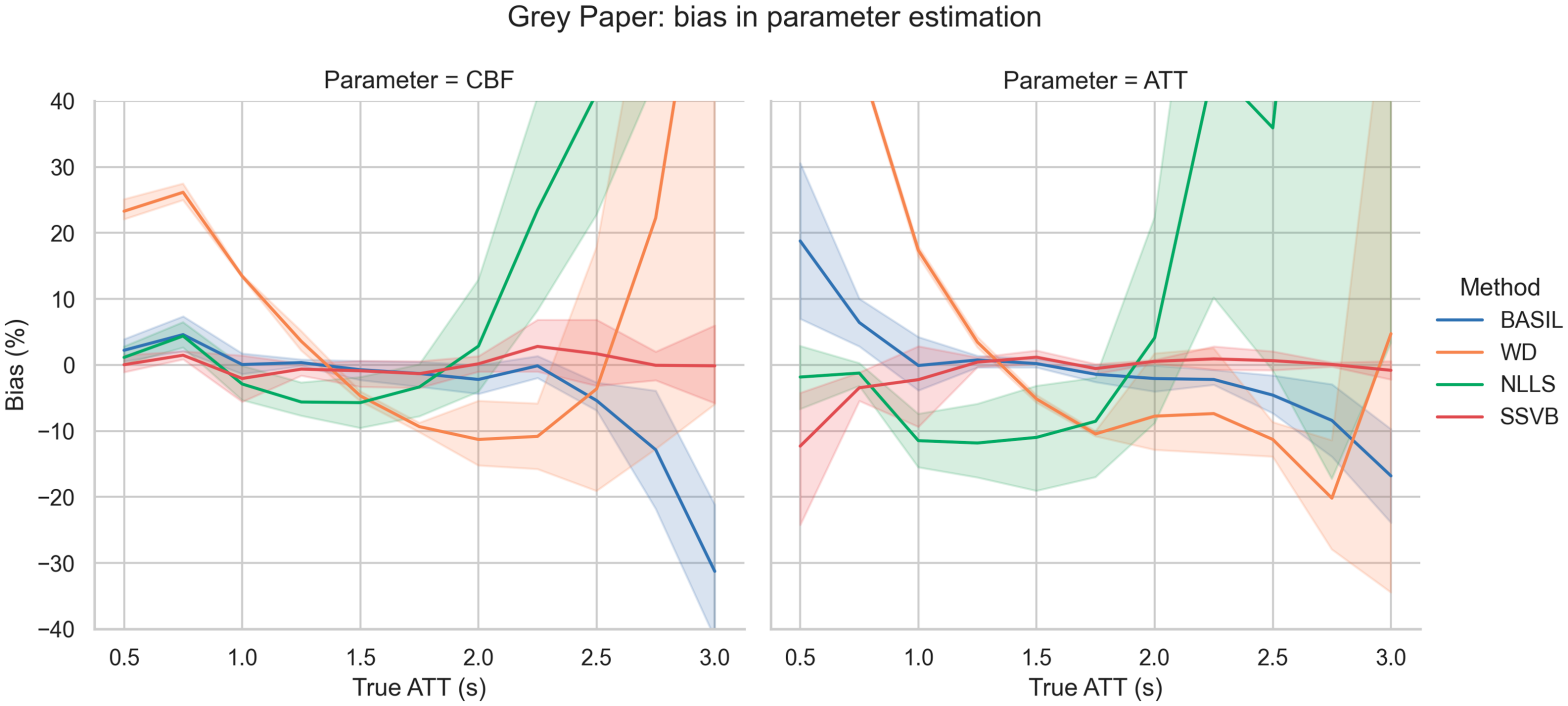
Bias in estimation of ground truth CBF (left) and ATT (right) as a function of ground truth ATT. The fan around each line represents variation across different SNR levels. SSVB’s estimates showed the lowest bias across the full range of simulation parameters. The *y*-axis has been restricted for clarity; the full range of results is given in Supplementary Figure 1. This figure is also re-drawn as Supplementary Figure 3 with noise SD on the *x*-axis to enable bias to be visualised as a function of noise.

Figure 2 shows bias in estimation of ground truth parameters from simulated data using the grey paper sequence. Across the full range of true ATT and SNR levels, SSVB’s estimates showed consistently low bias (always less than 12% averaged across noise levels) in both CBF and ATT. The most substantial bias of −12% was observed in ATT for true ATT = 0.5 s. BASIL produced similar results to SSVB for true ATT between 1.0 s and 2.5 s but showed greater bias outside this range (19% in ATT for true ATT = 0.5 s and − 30% in CBF for true ATT = 3.0 s). WD showed large positive bias for true ATT < 1.5 s (e.g., 20% in ATT at true ATT = 1.0 s) and NLLS showed large positive bias for true ATT > 2 s (e.g., 30% in ATT at true ATT = 2 s).

**Figure 3:**
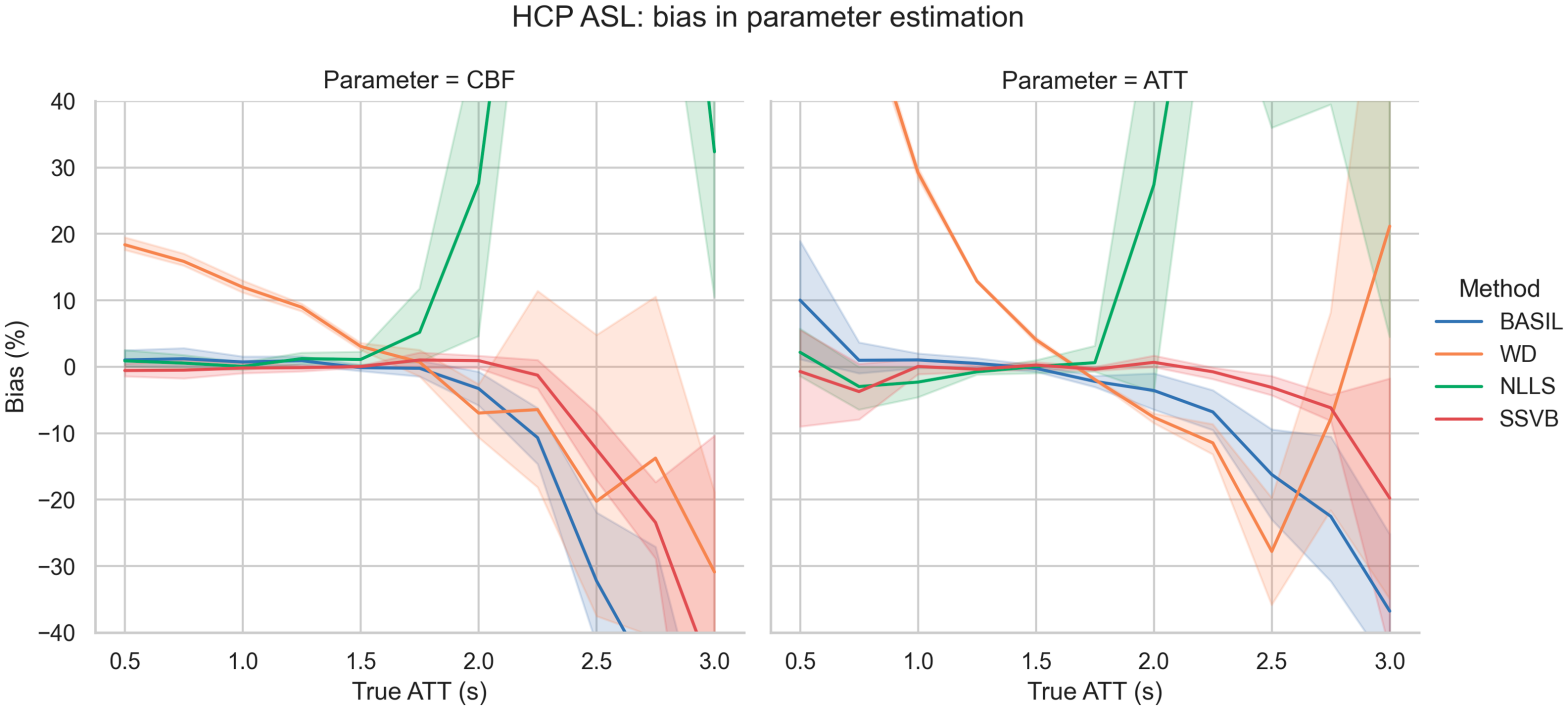
Bias in estimation of ground truth CBF (left) and ATT (right) as a function of ground truth ATT. The fan around each line represents variation across different SNR levels. SSVB’s estimates showed the lowest bias across the full range of simulation parameters, though performance deteriorated for true ATT > 2.5 s. The *y*-axis has been restricted for clarity; the full range of results is given in Supplementary Figure 2. This figure is also re-drawn as Supplementary Figure 4 with noise SD on the *x*-axis to enable bias to be visualised as a function of noise.

Figure 3 shows bias in estimation of ground truth parameters from simulated data using the HCP ASL sequence. For true ATT < 2.5 s, SSVB’s estimates showed consistently low bias (less than 13% in both CBF and ATT); BASIL performed similarly for true ATT < 2.0 s. WD showed large positive bias for true ATT < 1.5 s (e.g., 30% in ATT at true ATT = 1.0 s) and NLLS showed large positive bias for true ATT > 2.0 s (e.g., 25% in ATT at true ATT = 2.0 s).

### HCP ASL data

**Figure 4:**
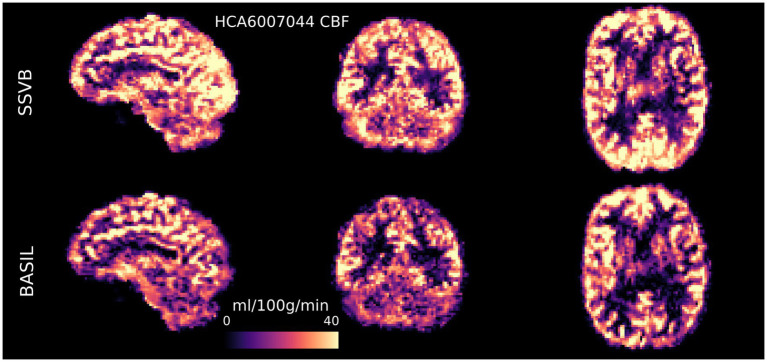
CBF maps (calibrated to ml/100 g/min) for subject HCA6007044 demonstrated the ability for high spatial resolution ASL to reveal fine anatomical detail in perfusion. There was broadly high agreement between SSVB and BASIL, though SSVB estimated slightly higher perfusion in posterior regions, visible for example in the sagittal view of the occipital lobe.

**Figure 5:**
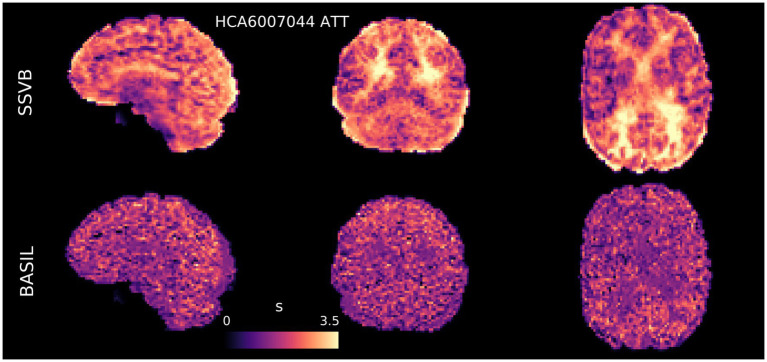
ATT maps (s) for subject HCA6007044 demonstrated substantial differences between the two methods: SSVB’s estimates were both less noisy and covered a wider range of ATT values. ATT estimated by SSVB in posterior WM was substantially higher than BASIL.

Figures 4, 5 show CBF and ATT maps produced by SSVB and BASIL for a single subject of the HCP ASL dataset. Whilst there was high similarity in the CBF maps (both exhibiting anatomical detail consistent with the high spatial resolution of the data), SSVB’s ATT map showed a much wider range of values, with notably longer ATT in white matter (WM) or watershed regions.

**Figure 6:**
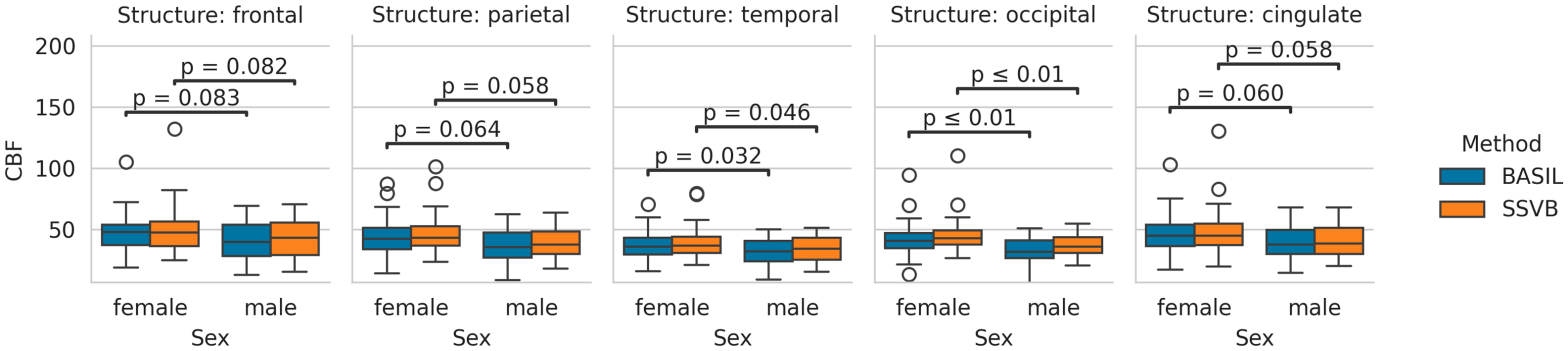
Distributions of cortical CBF estimates produced by each method, separated by sex. Both methods produced broadly similar estimates in all lobes, and both methods produced higher CBF estimates in females than males.

Figure 6 shows the distribution of cortical CBF estimates produced by each method across the dataset. No significant differences in CBF were observed between methods (comparisons given in Supplementary Table 1). Both methods found females to have higher CBF by approximately 5–8 mL/100 g/min, though this was significant only in the occipital lobe (comparisons are given in Supplementary Table 2).

**Figure 7:**
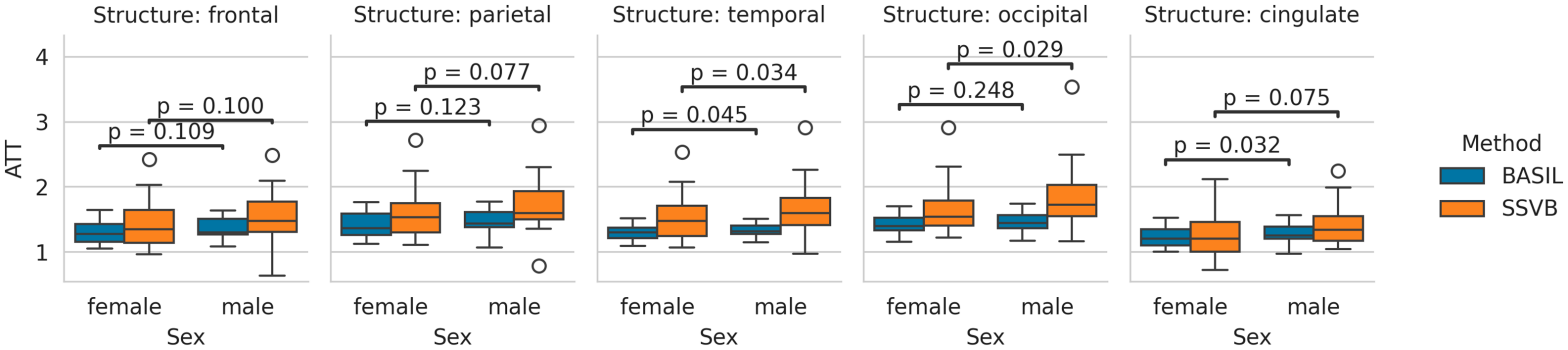
Distributions of cortical ATT estimates produced by each method, separated by sex. In all cortical lobes, the estimates produced by SSVB covered a wider range of values than BASIL. For both methods, males tended to have longer ATT than females; the difference was more pronounced for SSVB.

Figure 7 shows the distribution of cortical ATT estimates produced by each method. Across all cortical lobes, SSVB produced estimates covering a wider range of values than BASIL. Comparing methods within individuals, SSVB’s ATT estimate was significantly higher in all lobes except the cingulate (comparisons are given in Supplementary Table 4). For both methods, males were observed to have longer ATT than females but these differences were not significant (comparisons are given in Supplementary Table 5).

**Figure 8:**
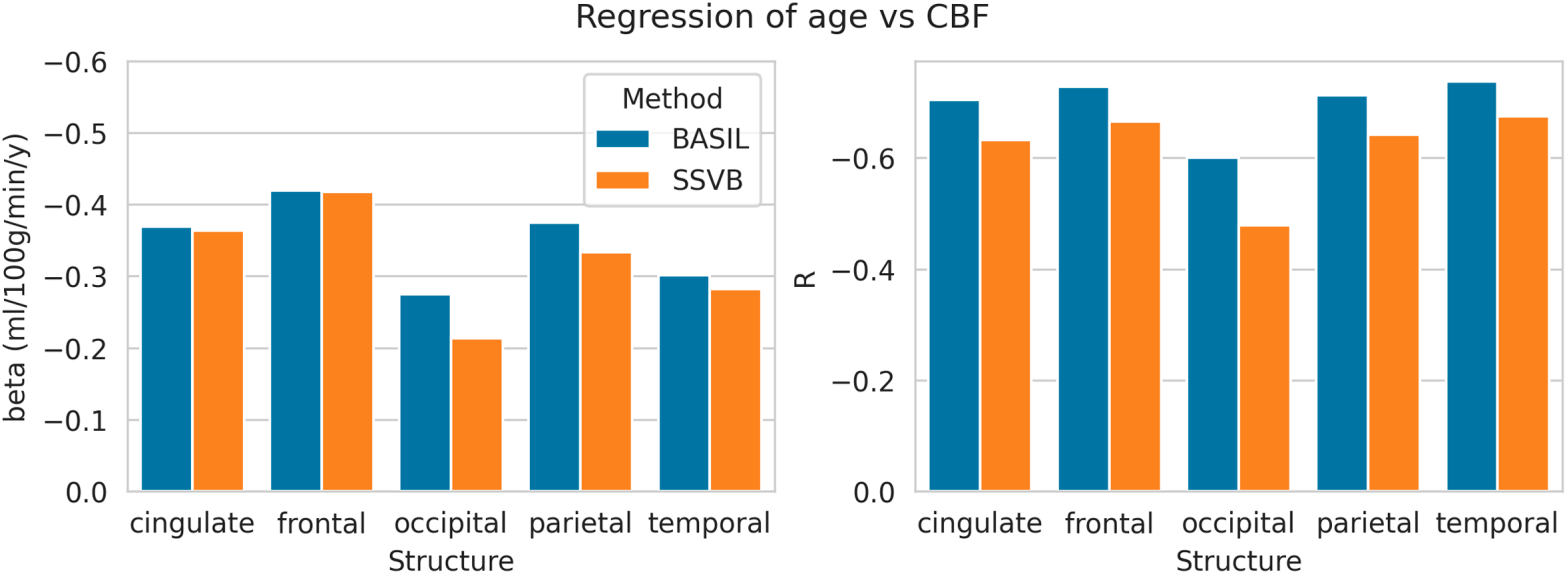
Linear regression coefficients of CBF (dependent) against age (independent) within each cortical structure. Numerical values used to draw this plot are given in Supplementary Table 3.

Figure 8 shows linear regression coefficients for CBF against age (all regressions were significant). In all cortical lobes, SSVB revealed a shallower (less negative) gradient and weaker correlation coefficient than BASIL, though the differences were not very substantial (for example, −0.27 vs. −0.21 in the occipital lobe). Numerical values are reported in Supplementary Table 3.

**Figure 9:**
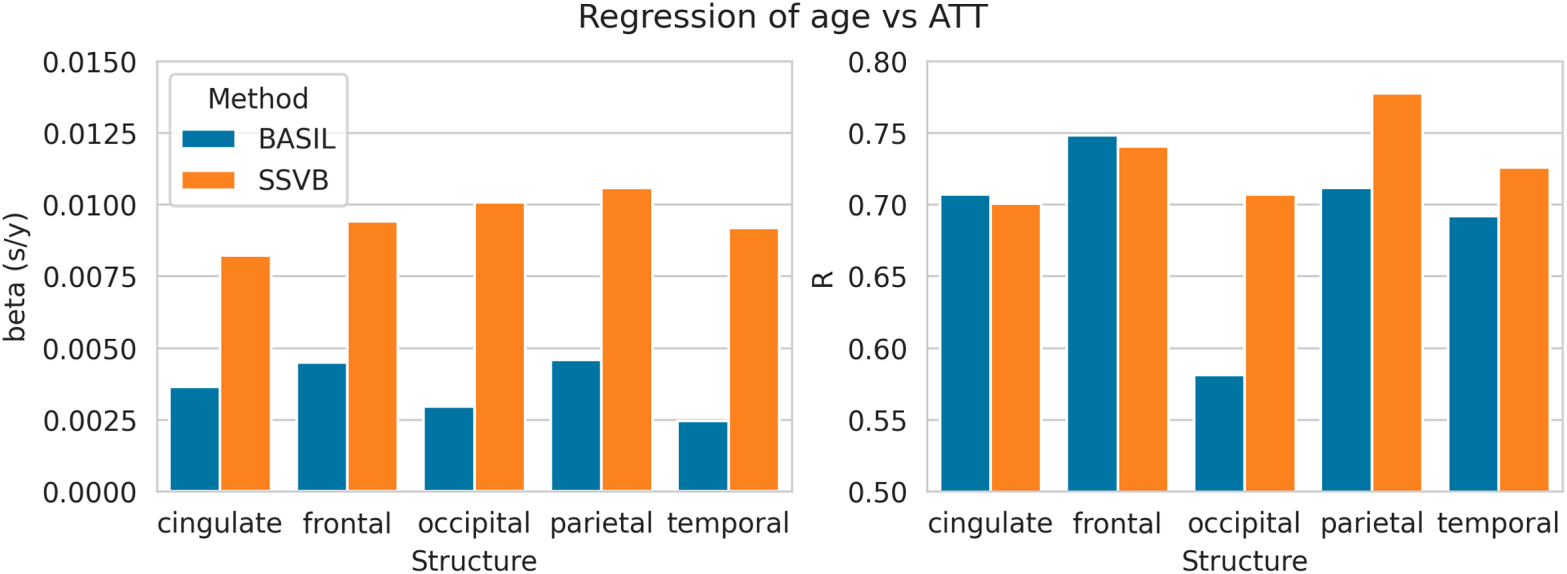
Linear regression coefficients of ATT (dependent) against age (independent) within each cortical structure. Numerical values used to draw these plots are given in Supplementary Table 6.

Figure 9 shows linear regression coefficients for ATT against age (all regressions were significant). In all cortical lobes, SSVB revealed a steeper relationship between age and ATT (the gradient was 2–3 times larger than for BASIL) with an approximately equal or larger correlation coefficient (particularly in the occipital, parietal and temporal lobes). Numerical values are reported in Supplementary Table 6.

**Figure 10:**
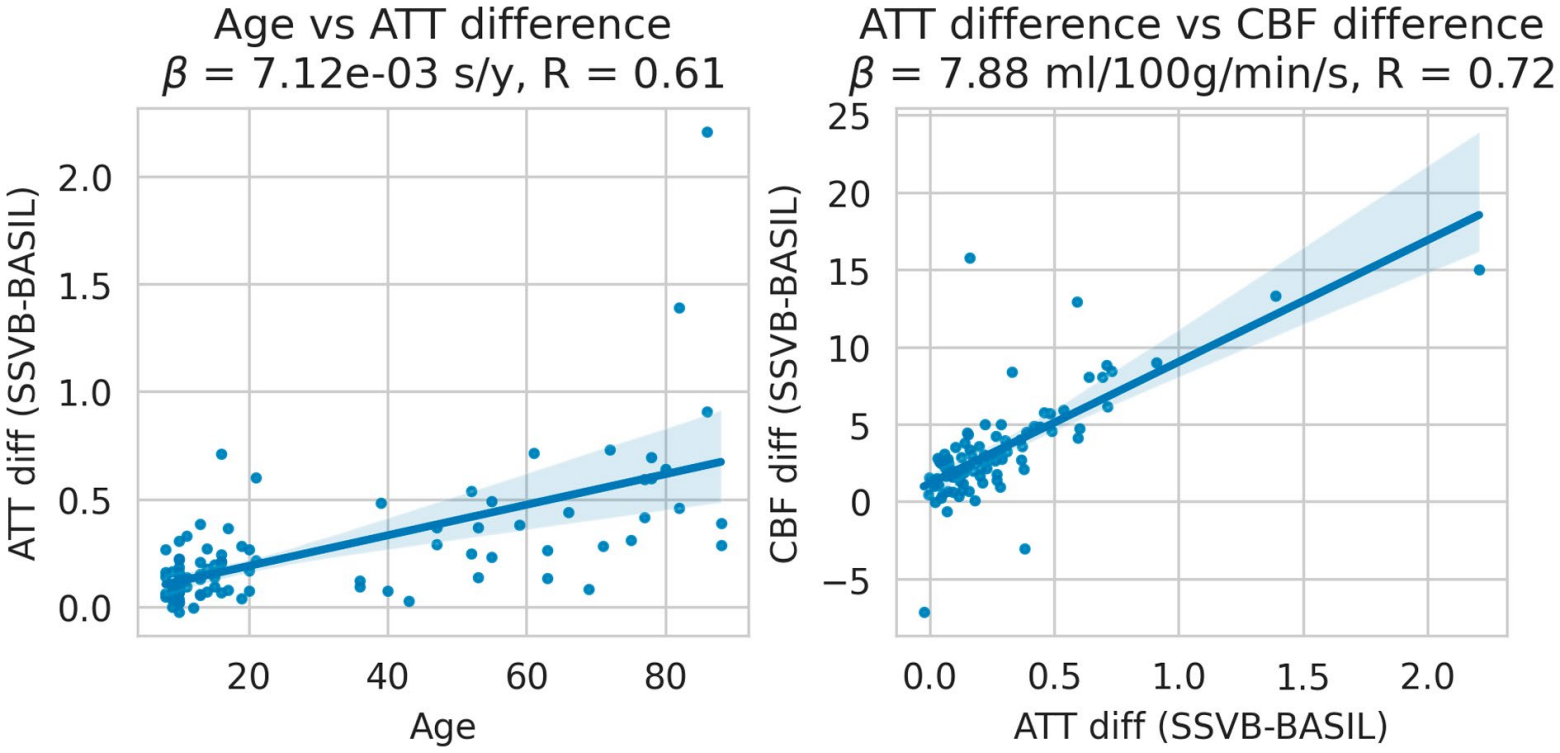
Linear regressions of methodological ATT differences (SSVB-BASIL) against age (left panel); and methodological ATT differences against CBF differences (right panel). Both panels are drawn for the occipital lobe only and both regressions were significant. ATT differences were observed to increase with age with reasonable correlation (0.61), such that SSVB estimated approximately 0.6 s higher ATT in 80-year-old subjects. These ATT differences correlated with CBF differences between the methods (*R* = 0.72), where are longer SSVB ATT estimated also implied a higher CBF estimate.

Figure 10 shows the interaction between age and methodological differences for ATT and CBF, restricted to the occipital lobe (which is most posterior and therefore typically has extended ATT). Methodological ATT differences were found to increase with age, such that SSVB estimated longer ATT in elderly subjects with a correlation of 0.61. Subject-wise ATT differences were in turn found to correlate (*R* = 0.72) with CBF differences such that longer ATT estimates were associated with higher CBF estimates.

## Discussion

Simulation results showed that SSVB was the most accurate and robust inference method over the range of simulation parameters investigated (Figures 2, 3). Particularly notable was the extra accuracy for ground truth ATT > 2.0 s, and consistency across SNR levels. These results indicate that SSVB is better suited to subjects with long ATT and low CBF (equivalent to low SNR), which is often the case for elderly cohorts or in neurodegenerative disease.

Results from HCP ASL data demonstrated the benefits of SSVB’s capabilities in ATT estimation. SSVB’s ATT maps were visibly less noisy than BASIL’s (Figure 5) and revealed expected anatomical features such as watershed regions or elongated ATT in WM. In agreement with previously reported results, both SSVB and BASIL’s ATT estimates increased with age, though the slope of the relationship was 2–3 times greater with SSVB than BASIL (Figure 9). Coupled with the smaller regression slope observed for SSVB between CBF and age (Figure 8), and the mapping of methodological ATT differences to CBF differences (Figure 10), this suggests that the extent of CBF decrease with age which has been previously reported is at risk of being overestimated if ATT is not accurately estimated.

Addressing between-method differences specifically, differences in ATT were most pronounced in elderly HCP ASL subjects (Figure 10), which is consistent with prior knowledge that ATT is elongated in elderly subjects, and consistent with the simulation results which revealed greater methodological differences for true ATT > 2 s (Figure 3). Potential sex differences in ATT were observed (Figure 7; Supplementary Table 4), though the number of subjects in the dataset was insufficient to reach statistical significance. If confirmed on a larger dataset, these would be an interesting addition to previously-reported sex differences in CBF (Leidhin et al., 2021).

Finally, the visual quality of the HCP ASL parameter maps demonstrated the potential of high spatial resolution ASL. Although ASL is often acquired at low spatial resolution to mitigate low SNR, the quality of SSVB’s maps showed that sophisticated perfusion estimation methods can “look through” noise to recover anatomical details that would otherwise be lost at low spatial resolution. This observation is consistent with the robust performance of SSVB on low SNR simulation data, and echoes previous work investigating the feasibility of high-resolution ASL (Kashyap et al., 2024).

Future work could investigate whether the extra flexibility afforded by SSVB can be exploited advantageously. For example, though MRI acquisition noise is routinely approximated as a zero-mean Gaussian distribution, a Rician distribution is theoretically more appropriate, and could be implemented in SSVB.

## Conclusion

SSVB is a new method for estimating perfusion from multiple-timepoint ASL data that offers increased accuracy and robustness compared to existing methods. Simulations show that the gains in performance over existing methods are greatest for true ATT > 2 s, which is pertinent for the study of elderly or potentially diseased populations. When run on high spatial resolution HCP ASL data, SSVB produced ATT maps with low noise and expected anatomical features such as watershed regions.

## Data Availability

The original contributions presented in the study are included in the article/Supplementary material, further inquiries can be directed to the corresponding author.
