# Supplementary material for: Stochastic variational inference improves quantification of multiple timepoint arterial spin labelling perfusion MRI
Source: Front Neurosci. 2025 Feb 4;19:1536752. doi: 10.3389/fnins.2025.1536752 (PMC11832661; doi:10.3389/fnins.2025.1536752)
Supplement: Supplementary file 4 [file Table_1.DOCX]

**Stochastic variational inference improves quantification** **of multiple timepoint arterial spin labelling perfusion MRI**

# Supplementary material

## Simulation results


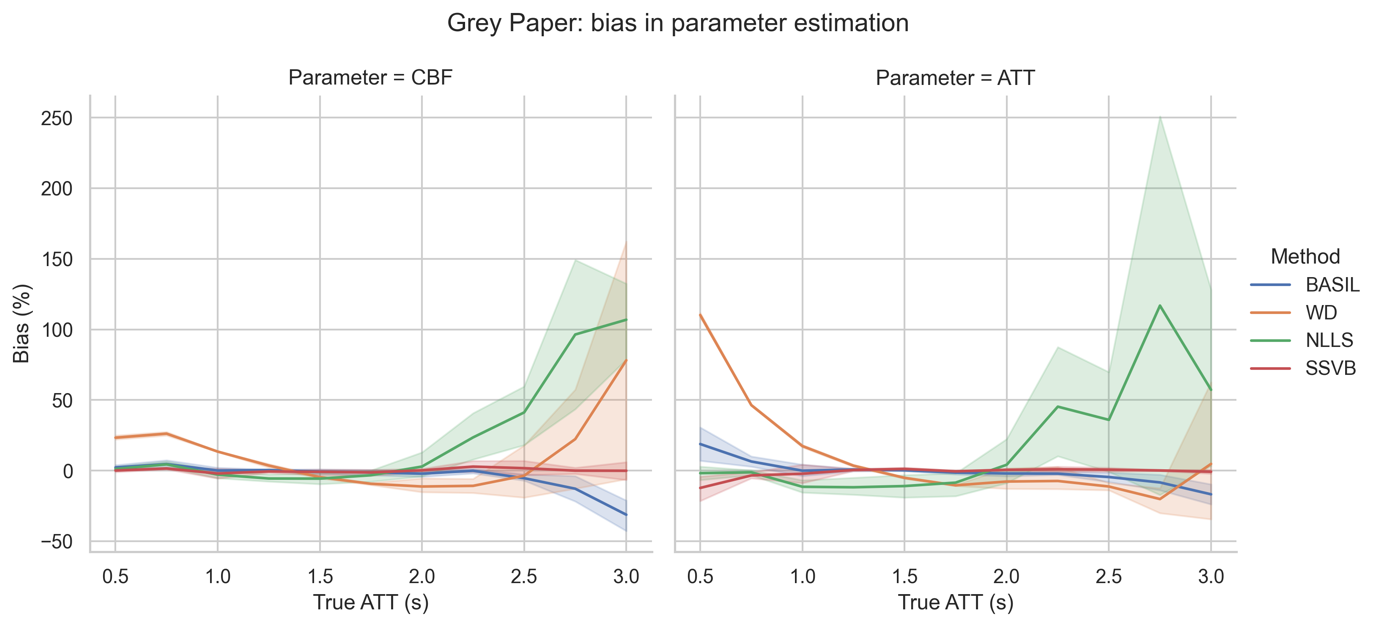


Supplementary Figure 1 Bias in estimation of ground truth CBF (left) and ATT (right) as a function of ground truth ATT for simulated grey paper sequence data. The fan around each line represents variation across different SNR levels.


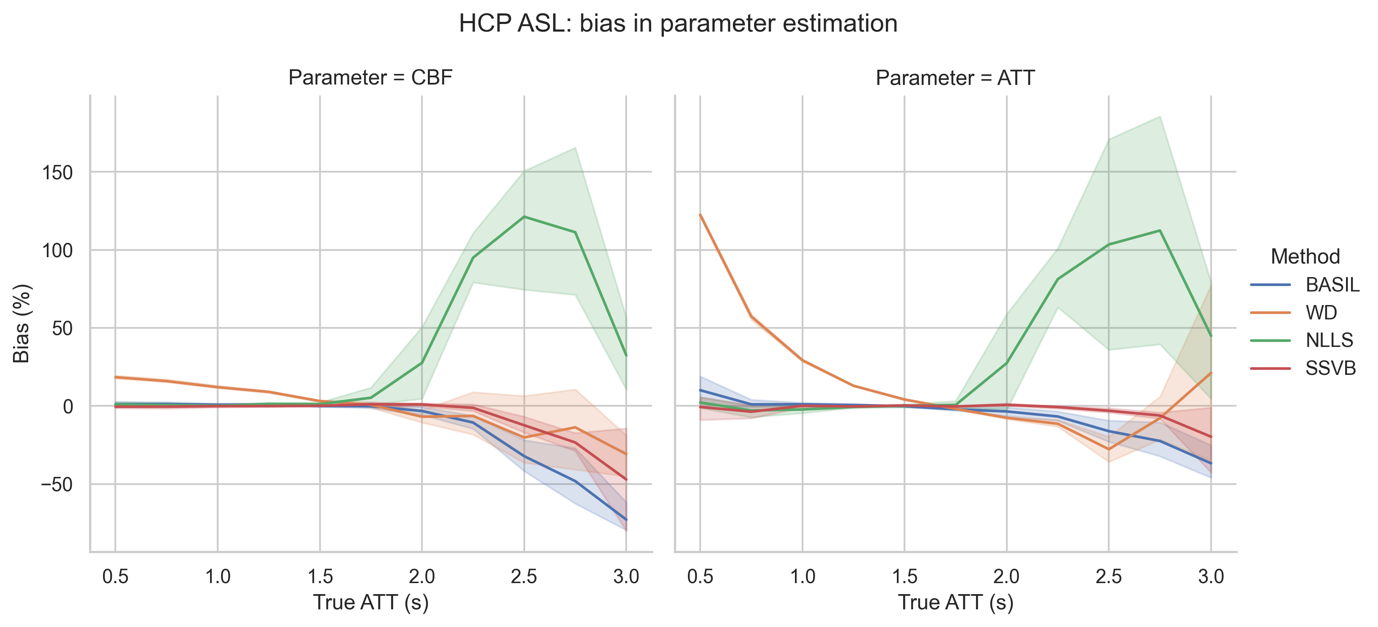


Supplementary Figure 2 Bias in estimation of ground truth CBF (left) and ATT (right) as a function of ground truth ATT for simulated HCP ASL sequence data. The fan around each line represents variation across different SNR levels.


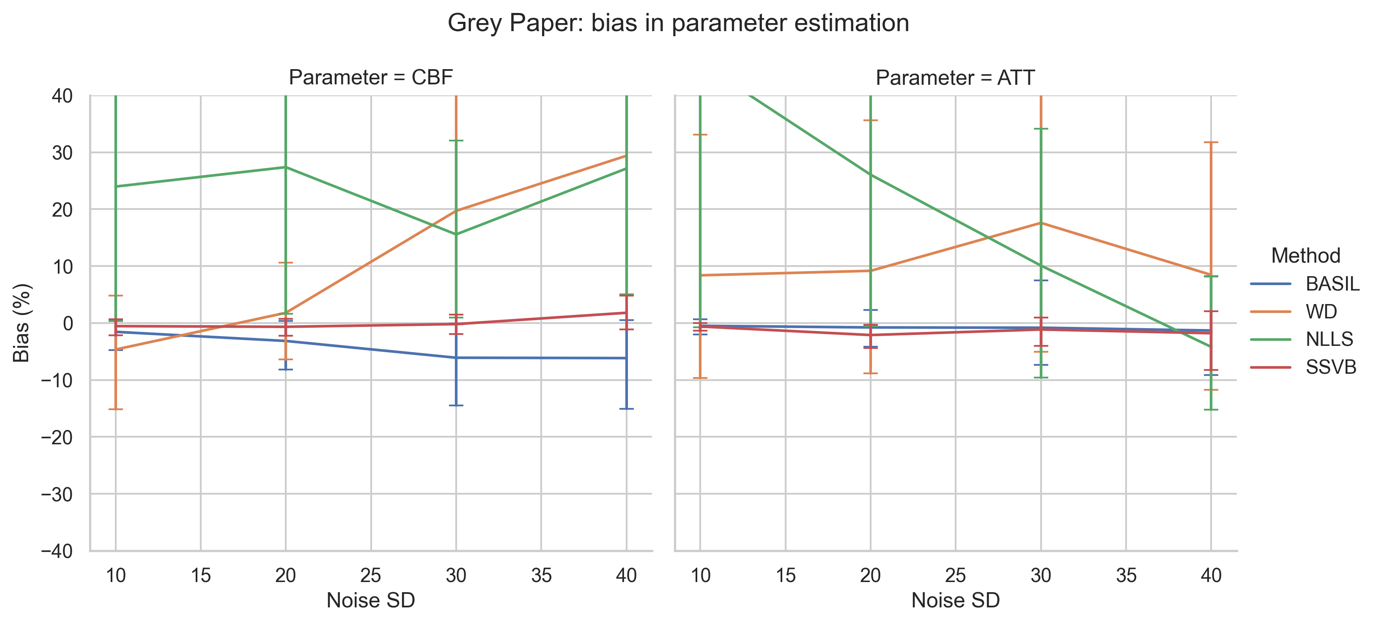


Supplementary Figure 3 Bias in estimation of ground truth CBF (left) and ATT (right) as a function of noise SD for simulated grey paper sequence data. The error bars represent variation across true ATT. For both CBF and ATT, SSVB’s bias was closest to zero across all noise levels. BASIL’s bias was close to zero in ATT but notably worse in CBF at higher noise levels (e.g., -7% at noise SD = 40).


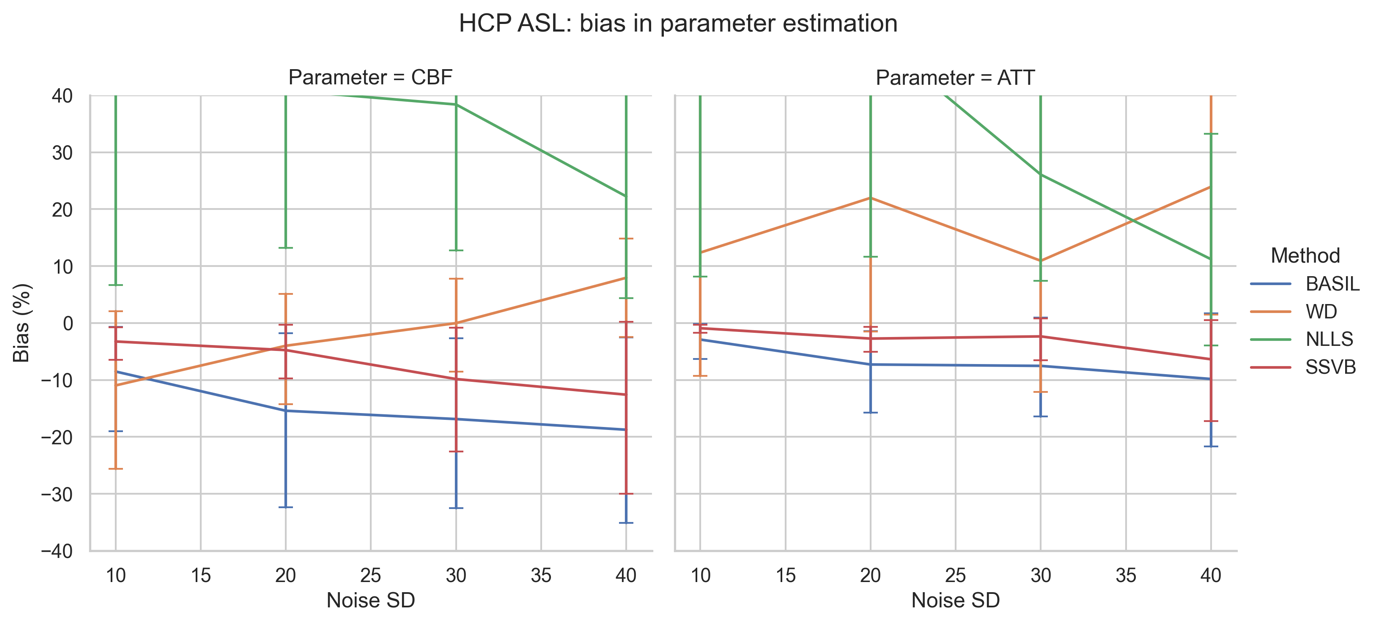


Supplementary Figure 4 Bias in estimation of ground truth CBF (left) and ATT (right) as a function of noise SD for simulated HCP ASL sequence data. The error bars represent variation across true ATT. For both CBF and ATT, SSVB’s bias was closest to zero across all noise levels, and consistently smaller than BASIL.

## HCP ASL tables

| **Structure** | **∆ CBF (SSVB-BASIL)** | **t** | **p** |
| --- | --- | --- | --- |
| **frontal** | 1.68 | 0.70 | 0.49 |
| **parietal** | 2.82 | 1.35 | 0.18 |
| **temporal** | 1.92 | 1.16 | 0.25 |
| **occipital** | 3.29 | 1.82 | 0.07 |
| **cingulate** | 1.42 | 0.64 | 0.52 |

Supplementary Table 1 Methodological differences in CBF estimates. Significance was not reached in any cortical structure.

|  | **∆ CBF (F-M)** | | **t** | | **p** | |
| --- | --- | --- | --- | --- | --- | --- |
| **Method** | **BASIL** | **SSVB** | **BASIL** | **SSVB** | **BASIL** | **SSVB** |
| **Structure** |  |  |  |  |  |  |
| **cingulate** | 5.641 | 6.200 | 1.935 | 1.994 | 0.056 | 0.049 |
| **frontal** | 5.707 | 6.243 | 1.750 | 1.798 | 0.084 | 0.076 |
| **occipital** | 8.339 | 7.840 | 3.428 | 3.368 | 0.001 | 0.001 |
| **parietal** | 5.555 | 5.633 | 1.890 | 1.958 | 0.062 | 0.053 |
| **temporal** | 4.984 | 4.746 | 2.193 | 2.075 | 0.031 | 0.041 |

Supplementary Table 2 Sex differences in CBF detected by each method. Whilst the group mean was always higher for females, significance was reached only in the occipital lobe for both methods.

|  | **beta (ml/100g/min/y)** | | **p** | | **R** | |
| --- | --- | --- | --- | --- | --- | --- |
| **Method** | **BASIL** | **SSVB** | **BASIL** | **SSVB** | **BASIL** | **SSVB** |
| **Structure** |  |  |  |  |  |  |
| **cingulate** | -0.37 | -0.36 | < 1e-10 | < 1e-10 | -0.70 | -0.63 |
| **frontal** | -0.42 | -0.42 | < 1e-10 | < 1e-10 | -0.73 | -0.67 |
| **occipital** | -0.28 | -0.21 | 3.84e-10 | 1.76e-06 | -0.60 | -0.48 |
| **parietal** | -0.37 | -0.33 | < 1e-10 | < 1e-10 | -0.71 | -0.64 |
| **temporal** | -0.30 | -0.28 | < 1e-10 | < 1e-10 | -0.74 | -0.68 |

Supplementary Table 3 Linear regressions of CBF against age. All regressions were significant, showing an approximately equal rate of CBF decline with age for each method, with reasonable correlation coefficients. The largest difference in rate of CBF decline was observed in the occipital lobe (-0.28 for BASIL, -0.21 for SSVB).

| **Structure** | **∆ ATT (SSVB - BASIL)** | **t** | **p** |
| --- | --- | --- | --- |
| **frontal** | 0.14 | 3.44 | <1e-3 |
| **parietal** | 0.20 | 4.63 | <1e-3 |
| **temporal** | 0.24 | 6.56 | <1e-3 |
| **occipital** | 0.27 | 6.31 | <1e-3 |
| **cingulate** | 0.05 | 1.42 | 0.16 |

Supplementary Table 4 Methodological differences in ATT estimates. SSVB estimated a longer ATT in all lobes; significance was reached in all structures except the cingulate.

|  | **∆ ATT (F-M)** | | **t** |  | **p** |  |
| --- | --- | --- | --- | --- | --- | --- |
| **Method** | **BASIL** | **SSVB** | **BASIL** | **SSVB** | **BASIL** | **SSVB** |
| **Structure** |  |  |  |  |  |  |
| **cingulate** | -0.063 | -0.119 | -2.205 | -1.793 | 0.030 | 0.077 |
| **frontal** | -0.055 | -0.120 | -1.661 | -1.662 | 0.100 | 0.100 |
| **occipital** | -0.034 | -0.177 | -1.163 | -2.144 | 0.248 | 0.036 |
| **parietal** | -0.057 | -0.137 | -1.591 | -1.771 | 0.115 | 0.081 |
| **temporal** | -0.041 | -0.153 | -2.089 | -2.121 | 0.040 | 0.037 |

Supplementary Table 5 Sex differences in ATT detected by each method. Whilst the group mean was always shorter for females (negative sign), SSVB’s estimate of the difference was approximately twice as large (e.g. -0.063 vs -0.119 in the cingulate). Significance was not reached in any cortical structure.

|  | **beta (s/y)** |  | **p** |  | **R** |  |
| --- | --- | --- | --- | --- | --- | --- |
| **Method** | **BASIL** | **SSVB** | **BASIL** | **SSVB** | **BASIL** | **SSVB** |
| **Structure** |  |  |  |  |  |  |
| **cingulate** | 0.0037 | 0.0082 | < 1e-10 | < 1e-10 | 0.71 | 0.70 |
| **frontal** | 0.0045 | 0.0094 | < 1e-10 | < 1e-10 | 0.75 | 0.74 |
| **occipital** | 0.0030 | 0.0101 | 1.913e-09 | < 1e-10 | 0.58 | 0.71 |
| **parietal** | 0.0046 | 0.0106 | < 1e-10 | < 1e-10 | 0.71 | 0.78 |
| **temporal** | 0.0025 | 0.0092 | < 1e-10 | < 1e-10 | 0.69 | 0.73 |

Supplementary Table 6 Linear regressions of ATT against age. All regressions were significant, and SSVB showed approximately twice the rate of increase of ATT with age (e.g. 0.0094 vs 0.0045 s/year in the frontal lobe). The correlation coefficient was approximately equal or larger for SSVB than BASIL, and always above 0.7.
